# Alternative methods of follow up in breast cancer: a systematic review of the literature

**DOI:** 10.1038/sj.bjc.6603771

**Published:** 2007-05-08

**Authors:** D A Montgomery, K Krupa, T G Cooke

**Affiliations:** 1 University Department of Surgery, Level 2, Queen Elizabeth Building, Glasgow Royal Infirmary, Glasgow G31 2ER, UK; 2 Department of Surgery, Royal Alexandra Hospital, Paisley, UK; 3 University Department of Surgery, Glasgow Royal Infirmary, UK

**Keywords:** breast cancer, follow up, recurrence, clinic

## Abstract

Regular clinical follow up after breast cancer is a common practice. Evidence from retrospective reviews casts doubt on the efficacy of this practice and the various guidelines for follow up show little concordance. Our aim was to investigate what alternative follow-up methods (including reduced frequency of visits) have been subjected to controlled trial and to establish what evidence exists from controlled trials to advise the guidelines. The study involved systematic review of the literature using MEDLINE, Embase, CancerLit, Web of Sciences and EBM reviews as data sources. Methods included reviewing all randomised controlled trials comparing different follow-up frequencies or comparing an alternative method with clinical follow up after breast cancer. All outcome measures addressed in the trials were analysed. Two trials compared frequency of traditional follow up. Five trials assessed alternative methods. All were of inadequate power or duration to establish ideal frequency of clinic visits or safety of alternative follow-up methods. Alternative follow up had no detrimental effect on satisfaction or outcome. Few trials have been conducted, all of which are underpowered to establish safety of reducing or replacing clinic visits. Alternative methods of follow up are acceptable to patients and may be associated with other benefits. Larger trials are required.

Regular follow up of patients after treatment for breast cancer is a common practice. Aims of follow up include early detection of treatable local recurrence, detection and treatment of side effects of therapy and psychological support in the aftermath of diagnosis and treatment (National Institute for Clinical Excellence, 2002).

Although it is generally felt that some form of follow up is required, it is not clear what form it should take. Multiple diagnostic interventions designed to detect metastatic disease are now no longer recommended (National Institute for Clinical Excellence, 2002; Grunfeld *et al*, 2005; Khatcheressian *et al*, 2006). These do not prolong survival and are considered to have a detrimental effect on the quality of life (Del Turco *et al*, 1994; The GIVIO investigators, 1994; Palli *et al*, 1999). The only investigation now conducted routinely is mammography, which has been shown to detect contralateral disease at an earlier stage than clinical examination alone (Mellink *et al*, 1991; Samant *et al*, 2001).

In addition to mammography, most clinicians recommend routine clinic visits for history and clinical examination. However, recommended frequency and duration of visits vary. The American Society of Clinical Oncology recommends clinical examination 3–6 monthly for 3 years, 6–12 monthly for a further 2 years and then annually (Khatcheressian *et al*, 2006). In contrast, the National Institute for Clinical Excellence (NICE) in England advocates follow up for only 2 or 3 years and gives no advice on how frequently visits should be conducted (National Institute for Clinical Excellence, 2002).

These marked variations in practice stem from a lack of evidence that routine clinical examination is of benefit. The NICE guidelines cite only one study, a retrospective review of 643 patients with median follow up of 3 years and 11 months, as evidence for their recommendations for routine follow up (Donnelly *et al*, 2001).

A recent systematic review designed to investigate long-term outcomes in the setting of routine clinical follow up failed to identify any trials comparing long-term results (survival/disease-free survival) in patients with clinically detected local recurrence *vs* symptomatic presentation of local recurrence (de Bock *et al*, 2004). The authors were unable to comment on whether routine clinical examination during follow up conferred any survival benefit (de Bock *et al*, 2004). However, they did report that routine clinics following the American Society of Clinical Oncology guidelines would detect only four asymptomatic recurrences in every 100 women over 10 years of follow up, and would lead to delayed diagnosis in two women who would notice problems but wait for their next clinic appointment (de Bock *et al*, 2004). A recent simulation model has also suggested that the survival benefit of follow up would be at best marginal (Jacobs *et al*, 2001).

It is clear that most retrospective reviews of routine clinic visits have not been helpful in establishing either the best schedule for such visits, or indeed whether such visits are necessary (de Bock *et al*, 2004). However, the available evidence does appear to suggest that the impact of routine follow up on survival will be at best marginal and may lead to delayed diagnosis of recurrence.

The optimal design to study this issue would be a randomised trial. Comparisons of different frequencies of visits to clinic or different durations of follow up would be of particular value in informing guidelines. Comparisons of traditional follow up with novel methods of care would help to establish whether traditional clinic visits were necessary. It is the intention of this review to identify any alternative methods of follow up, which has been proposed and subjected to randomised trial. In addition, we intend to establish whether there is any randomised, controlled evidence to suggest an ideal length or schedule of follow up in order to reconcile the various guidelines described above.

## MATERIALS AND METHODS

We searched MEDLINE, Embase, EBM reviews, including the Cochrane database, CancerLit and Web of Sciences for relevant studies in May 2006. Any study published between 1966 and present day was considered. The complete search string used is reproduced as Appendix A. The complete search string used for CancerLit is reproduced as Appendix B and for Web of Sciences as Appendix C. This initial search was conducted independently by authors DA Montgomery and K Krupa and titles were studied to assess which abstracts should be obtained. All abstracts obtained were read and considered independently by both DA Montgomery and K Krupa to assess whether the full-length article should be obtained. Sources of disagreement at this stage resulted in the full-length article being obtained. References of all full-length articles obtained were also searched for further relevant studies.

For all selected articles, MEDLINE, Embase, Web of Science and CancerLit were searched for articles, which had subsequently been published and had cited them.

### Selection criteria

Studies were included if they fulfilled the following criteria:

1. Patients included had been treated for primary operable breast cancer and were free of distant metastases outside the breast or axilla at the time of initial treatment.
2. The study was a randomised controlled trial comparing routine clinical and mammographic follow up with an alternative, or comparing different frequencies or durations of clinical follow up. Blinding of method of follow up to either clinicians or patients was considered unnecessary.

### Data extraction

Data were extracted by two investigators (DA Montgomery and K Krupa) independently. Topics included number of patients, percentage agreeing to randomisation, age, primary surgical treatment, study comparison, follow up schedule and duration of follow up.

Data were also extracted on the main outcome measures used and how these were determined. Outcome measures included patient satisfaction, quality of life, number of events (local recurrence, distant recurrence, significant adverse events and death) and economic analysis. Whether satisfaction and quality of life were measured in a validated manner was also recorded.

### Assessment of methodological quality

Methodological quality was also assessed independently by DA Montgomery and K Krupa by means of a predefined form. This form was derived from a number of sources, guided by a summary by Greenhalgh (1997) on the measurement of methodological quality in scientific trials and by the work of Altman and co-workers (Altman and Lyman, 1998) and Laupacis *et al* (1994). The results of this quality assessment are provided in Table 1.

## RESULTS

In all, 2248 titles were examined in MEDLINE, 944 in EMBASE, 225 in EBM reviews, 2882 in CancerLit and 331 in Web of Sciences. Five review, articles were also obtained in order to examine the references for further relevant studies (Anonymous, 1998; Collins *et al*, 2004; de Bock *et al*, 2004; Heys *et al*, 2005; Rojas *et al*, 2005). In total, 20 abstracts were obtained.

From these abstracts, six full-length articles were obtained, all of which were eligible for inclusion in the analysis (Grunfeld *et al*, 1996, 2006; Gulliford *et al*, 1997; Brown *et al*, 2002; Koinberg *et al*, 2004; Kokko *et al*, 2005). One abstract had been published as part of the proceedings of a scientific meeting and the data had not yet been published in full (Baildam *et al*, 2004). This abstract was also included. In total, we identified seven randomised controlled trials in the literature, all of which were eligible for inclusion.

### Study characteristics

The characteristics of all seven included trials are provided in Table 2.

Two trials compared follow up in hospital clinics with that provided by a general practitioner (Grunfeld *et al*, 1996, 2006), two compared traditional follow up with follow up merely on demand by contacting a breast care nurse (Brown *et al*, 2002; Koinberg *et al*, 2004) and one compared routine follow up by doctors with routine follow up by breast care nurses (Baildam *et al*, 2004). Two trials compared different frequencies of follow up within a traditional framework (Gulliford *et al*, 1997; Kokko *et al*, 2005).

### Methodological quality

As stated above, methodological quality was assessed by means of a predefined form. Methodological quality in general among these trials was good. However, one study (Baildam *et al*, 2004) scored poorly on this form as a result of being presented only in abstract form in the literature as part of the proceedings of an international meeting. The average score was 8.7 out of 13, or 10.7 if Baildam and co-workers are excluded.

Rating of methodological quality took into account intended outcome measures when assessing adequacy of sample size and follow-up duration. However, many of the trials had either inadequate numbers of patients or insufficient follow-up duration, or indeed both of these problems, for an analysis of long-term survival to be conducted.

### Detection of recurrence

Whether traditional clinical follow up is carried out 3 or 6 monthly makes no difference to the overall number of recurrences detected (Kokko *et al*, 2005). In 239 women followed up 3 monthly, there were 66 recurrences, compared with 57 recurrences among 233 women followed up 6 monthly. Local recurrence was not dealt with separately, however, and time to detection of recurrence was not analysed. It is unclear whether further increasing follow up interval to annual would have an effect on detection of local recurrence. One study attempted to address this issue, but recruited only 196 patients and analysed after a median of 16 months follow up, so had insufficient patient numbers and follow-up duration to conduct such analysis (Gulliford *et al*, 1997).

There was no difference in the number of recurrences between patients followed up by a physician and by a breast care nurse (seven *vs* six, respectively, from 525 patients followed up for an undisclosed time period) (Baildam *et al*, 2004). Time to recurrence was again not presented.

More local recurrences were detected by nurses during on-demand follow up than during routine visits in one trial (Koinberg *et al*, 2004). Nurses detected 12 recurrences from 133 patients compared with eight local recurrences detected by physicians from 131 patients. Again, these numbers are too small for this to be significant, and there was no difference in the number of patients diagnosed with metastatic disease. There were no significant differences between the groups in terms of time to event for any of the event types (time to locoregional recurrence, metastases or death) analysed (data not shown).

In the first of the trials dealing with hospital follow up compared with follow up in general practice, 296 patients were recruited, 148 in each type of follow up. There were similar numbers of locoregional recurrences in each group, with four in the general practice group and three in the hospital group. There were more than twice as many metastatic recurrences diagnosed in the hospital group (13 *vs* 6 in general practice, difference 4.7%, 95% confidence interval (CI) −0.8 to 10.3%) (Grunfeld *et al*, 1996). Of interest, while all the recurrences in the general practice group were detected by the general practitioner, 44% of the recurrences in the hospital follow up group were also diagnosed by the general practitioner initially (Grunfeld *et al*, 1996).

In the second of the hospital *vs* general practice follow up trials, there was again no significant difference in the proportion of women presenting with local or distant recurrences between the two groups (11.2% in general practice group compared with 13.2% in the hospital group, difference of 2.02%, 95% CI −2.13 to 6.16%). Time to recurrence detection is not provided (Grunfeld *et al*, 2006).

### Adverse clinical events

One trial addressed the issue of recurrence-related serious clinical events (SCEs) (Grunfeld *et al*, 2006). The authors postulate that early diagnosis of recurrence is likely to result in reduced rate of serious events related to recurrence, such as spinal cord compression from spinal metastases etc. They found no difference in rate of SCEs between hospital and general practice follow up (3.7% of patients *vs* 3.5%, respectively, difference 0.19%, 95% CI −2.26 to 2.65%).

### Survival

There was no difference between physician follow up or follow up on demand by a breast care nurse in number of deaths, with 14 deaths from 131 physician followed up patients compared with 14 deaths from 133 patients followed up by a nurse. Overall survival on Kaplan–Meier curves was similar for the two groups also (data not shown) (Koinberg *et al*, 2004).

One study showed a slight excess in mortality in hospital follow up compared with general practice (Grunfeld *et al*, 1996). The difference was not further analysed as the numbers were small, with only two deaths in the general practice group and seven in the hospital group from 148 patients in each cohort. Moreover, this difference did not exist over longer follow up in a much larger cohort of 968 women in a subsequent trial of hospital follow up compared with general practice by the same authors (Grunfeld *et al*, 2006). In this latter trial, there were 29 deaths in the general practice group and 30 in the hospital group.

### Satisfaction

Willingness to participate (percentage randomisation) is provided in Table 2.

Two studies looked at satisfaction with an alternative method of follow up. One used a structured interview technique (Brown *et al*, 2002), whereas the other employed a questionnaire previously validated by the authors (Koinberg *et al*, 2004). Both reported high levels of satisfaction with follow up in both study and control groups, with no difference in satisfaction between the study and control groups in either trial. In the patient-initiated follow-up trial by Brown *et al*, there were differences in what patients perceived as advantages of different methods of follow up, with more women describing routine clinic visits as reassuring (*χ*^2^:27.63, *P*<0.000, 1 df), whereas more men described on-demand follow up as convenient (*χ*^2^:17.354, *P*<0.000, 1 df) (Brown *et al*, 2002).

Within a traditional follow-up schedule, frequency can be reduced with no loss of satisfaction (Gulliford *et al*, 1997). Moreover, similar numbers in the high- and low-frequency groups will express a desire for lower- or higher-frequency visits than they received during the trial (Gulliford *et al*, 1997).

In the trial by Baildam *et al* (2004), the Fallowfield Satisfaction with Consultation Questionnaire revealed that women were significantly more satisfied with their consultation with a nurse than those seen by a doctor (*P*<0.001).

### Quality of life

Several trials looked at the issue of quality of life measured by validated tools such as the hospital anxiety and depression scale (HADS) (Grunfeld *et al*, 1996, 2006; Brown *et al*, 2002; Baildam *et al*, 2004; Koinberg *et al*, 2004), European Organisation for Research and Treatment of Cancer (EORTC) QLQ-C30 and BR23 (Grunfeld *et al*, 1996; Brown *et al*, 2002) and the Medical Outcomes Study Short Form 36-item General Health Survey (SF-36) (Grunfeld *et al*, 1996, 2006). None of these trials reported any differences in any of the scales between control and study groups at any stage in the respective trials.

Although there were no differences in quality of life between control and study groups in any of the trials, one group did report that nurses were better at recognising psychological distress than doctors (Baildam *et al*, 2004). From a cohort of 525 women, 86 patients had a psychological distress indicated by HADS scores, 49 seen by a nurse and 37 by a doctor. Whereas nurses failed to recognise this in 47%, doctors missed distress in 92% of these women.

### Economic analyses and workload concerns

Unsurprisingly, reducing the frequency of clinic follow up is shown to reduce the cost of follow up, with a mean cost of follow up of 1656€ in the 3 monthly follow up group compared with 1050€ per patient in the 6 monthly follow up group over 4.2 years of follow up (Kokko *et al*, 2005). There was no increase in the number of phone calls or visits to general practitioners as a result of reduced frequency of follow up visits (Gulliford *et al*, 1997).

Another study revealed no advantage of breast care nurse follow up over follow up by clinicians in terms of cost (data not shown) (Baildam *et al*, 2004). This finding was attributed to the fact that nurses spent longer with individuals than doctors, and that very senior nursing staff were required.

Grunfeld *et al* (1996) subsequently published the results of an economic analysis of follow up in hospital compared with follow up by General practitioner (Grunfeld *et al*, 1999). General practice patients were seen significantly more frequently and each visit was significantly longer during the 18 months of the trial than were hospital patients. In addition, while overall numbers of tests were similar, general practitioners requested significantly more blood tests, mammograms and chest X-rays than specialists. Overall, however, the cost to the health service was lower for general practice follow up than for follow up in hospital (Grunfeld *et al*, 1999).

Follow up on demand by breast care nurses results in significantly fewer clinical contacts. There were 450 fewer visits to the physician and only 177 more phone calls, and 88 more visits to the nurse in one study, so that there were 21% more primary contacts in the physician group (Koinberg *et al*, 2004). There were more mammograms in the nurse-led on-demand group, however.

## DISCUSSION

Numerous guidelines exist for the follow up of breast cancer, with little concordance between them. In the UK, The National Institute for Clinical Excellence (NICE) recommends 2–3 years of follow up then discharge to general practice (National Institute for Clinical Excellence, 2002). The British Association of Surgical Oncology (BASO) suggests discharge at 5 years (The Association of Breast Surgery @ BASO and Royal College of Surgeons of England, 2005). In sharp contrast, the American Society of Clinical Oncology recommends 3–6 monthly visits for 5 years, followed by annual visits indefinitely (Khatcheressian *et al*, 2006), and the Canadian Steering Committee similarly advocates indefinite follow-up visits for clinical examination (Grunfeld *et al*, 2005). But which of these organisations is correct?

Routine clinical examination after treatment for breast cancer is an inefficient way of detecting recurrent disease (de Bock *et al*, 2004). Intuitively, more frequent clinic visits should lead to earlier detection of recurrence and improved survival at the cost of reduced efficiency. However, there is no evidence in the literature that this is the case.

Only two randomised controlled trials have attempted to address the frequency of follow up (Gulliford *et al*, 1997; Kokko *et al*, 2005). Neither provided clear answers. Kokko *et al* (2005) demonstrated that 3 and 6 monthly follow ups are equivalent in terms of event detection, but made no comment on survival and did not separate locoregional from distant recurrence. The trial by Gulliford *et al* (1997)was insufficiently powered to comment on the safety of reducing follow up to annual visits. No randomised trials have looked at the issue of when patients should be discharged from clinic.

Five randomised controlled trials exist comparing traditional follow up with some alternative (Grunfeld *et al*, 1996, 2006; Brown *et al*, 2002; Baildam *et al*, 2004; Koinberg *et al*, 2004). However, the authors have largely sought to measure outcomes such as acceptability to patients, satisfaction, quality of life etc. Most of the trials have resultantly been underpowered or of inadequate duration to address the issue of survival.

Those investigators who have attempted to address the issue of survival have shown no disadvantage to follow up without the hospital setting, either by general practitioner (Grunfeld *et al*, 2006) or on demand by a specialist nurse (Koinberg *et al*, 2004). In one of these trials, almost 1000 women were followed until 4 and a half years after treatment and survival, locoregional and distant recurrence detection and incidence of recurrence-related SCEs were identical in both arms (Grunfeld *et al*, 2006). However, event rates will be small even in such a large cohort of women. In 1312 women in Edinburgh followed for a median duration of 10 years, only 110 treatable locoregional recurrences occurred, with only 15 diagnosed by clinical examination, the rest being either symptomatic or mammographically detected (personal communication). Therefore, despite the large recruitment and reasonable duration of follow up in the trial by Grunfeld and co-workers, even this trial was insufficiently powered to show a difference in outcome related to follow up.

Two trials have demonstrated a trend to more locoregional recurrences being detected by alternative follow up methods (Grunfeld *et al*, 1996; Koinberg *et al*, 2004). Both trials were of a limited duration. With longer follow up, the question of whether this represents earlier detection of locoregional recurrence during non-hospital based follow up, and whether this would result in improved long-term survival with these alternative methods of follow up, may have been answered. Certainly, large meta-analyses have reported that routine clinical follow up results in delayed diagnosis of locoregional recurrence in two-thirds of women who develop recurrence (de Bock *et al*, 2004).

None of the trials revealed any reduction in the quality of life or increase in levels of stress associated with alternative follow up (Grunfeld *et al*, 1996, 2006; Brown *et al*, 2002; Baildam *et al*, 2004; Koinberg *et al*, 2004). Moreover, alternative follow up methods appear to be acceptable to women, as evidenced by high randomisation rates. The alternative methods can be more cost effective and reduce workload at busy specialist clinics (Gulliford *et al*, 1997; Grunfeld *et al*, 1999). So why have so few investigators made any serious attempt to address this issue?

In correspondence published in response to the original trial by Grunfeld *et al* (1996), there was support from general practitioners for general practice follow up. However, there was an assertion from breast surgeons that clinical examination was central to the detection of locoregional recurrence, and that those conducting such examination should have some degree of specialisation (Dixon and Norman, 1996; Rainsbury, 1996). They warn that non-specialists may be less able to detect locoregional recurrence, especially in those who have had breast conserving therapy, or to detect metastatic disease when it presents with nonspecific symptoms (Rainsbury, 1996).

Rodger asserts that clinicians gain the experience they have in detecting recurrent disease by frequent examination of a range of normal. Reducing clinical follow up may result in de-skilling of clinicians, or at least reduced experience of those currently in training (Rodger, 1997). In addition, removing patients from routine follow up would have the effect that clinicians only see patients with problems. This may lead to reduced job satisfaction and the risk of subsequent burnout (Khatcheressian and Smith, 2006). While these are valid concerns, it does raise the question of whether we follow up well patients for their benefit or ours.

Whatever clinicians perceive as their reasons for continuing with clinical follow up, it is the perception we have of patients’ expectations that has done the most in reducing our exploration of alternative follow up methods. While the idea of coming back to specialist clinics less frequently is associated with a high degree of acceptance among women, as evidenced by a 93% randomisation to annual follow up in the trial by Gulliford *et al* (1997), the idea of non-specialist follow up is less acceptable. Randomisation rates for the two trials involving follow up by a non-specialist were 55 and 66.5% (Grunfeld *et al*, 1996, 2006). Morris *et al* (1992) reported that 81% of women in routine breast cancer follow up were reassured by the clinic and 76% preferred hospital clinics to being followed up in general practice. Similar findings have been reported by others (Kiebert *et al*, 1993; Paradiso *et al*, 1995; Renton *et al*, 2002).

When surveyed about their perceptions of cancer follow up, patients have expressed anger and distress about being discharged to their general practitioner (Maher *et al*, 1995). Patients viewed the hospital, and in particular diagnostic tests, specialist physicians and breast care nurses, as their best defence against recurrence. While they do report that there are negative points to coming to clinic – rushed consultations, long waiting times and lack of continuity of care – these are seen as an acceptable trade off for guarding against a relapse (Maher *et al*, 1995).

However, many women find follow up visits stressful. In one survey, almost half of the patients reported to be worried, anxious or frightened before appointments (Renton *et al*, 2002), and psychological stress is significantly higher among cancer patients a month before and on the day of clinic visits than 2 weeks after that (Kiebert *et al*, 1993). Up to 70% of breast cancer patients may suffer distress during routine clinical follow up (Paradiso *et al*, 1995).

None of the trials of alternative methods of follow up included here set out to measure the psychological stress of attending clinic. As a result, it is not clear whether alternative methods of follow up have less attendant stress. Replacing routine clinical follow up with some alternative does not appear to have a negative impact on patient psychological well being in general. In the studies, which reported on quality of life, there was no reduction in scores among patients in the trial group either compared with baseline or compared with the control patients (Grunfeld *et al*, 1996, 2006; Brown *et al*, 2002; Baildam *et al*, 2004; Koinberg *et al*, 2004). However, there was no improvement in the quality of life scores either.

## SUMMARY

The current recommendations for follow up are inconsistent (National Institute for Clinical Excellence, 2002; Grunfeld *et al*, 2005; Khatcheressian *et al*, 2006). This systematic review of the literature highlights the underlying reasons for the limitations in the recommendations.

There are no randomised trials in the literature with sufficient power to recommend an acceptable frequency or duration of follow up. Moreover, there are no randomised trials, which can confirm the safety of using alternative follow-up methods. Those studies, which have been conducted have not suggested that alternative methods are any less safe than routine clinical follow up (Grunfeld *et al*, 1996, 2006; Koinberg *et al*, 2004), but recruitment and duration are such that the only conclusion, which can be made is that the necessity for clinical examination and the safety of alternative follow up has not been proven.

Traditional routine clinic visits are an inefficient way of safeguarding against recurrent disease, and there is doubt as to whether they provide the ideal setting for providing psychological support to patients. Alternative follow-up methods are acceptable to patients, are associated with no reduction in the quality of life or increase in anxiety and may be conducted with significant economic and time savings.

More high-quality, randomised controlled trials in this area are required in order to establish how best to provide effective psychosocial support to patients after treatment of breast cancer, while at the same time maintaining adequate surveillance to detect those few treatable relapses which occur.

Clinicians involved in the care of breast cancer patients need to be clear about what their objectives are regarding follow up after breast cancer, both in their own minds and to their patients. Only then can we design and evaluate efficient follow-up programmes to meet those objectives, while at the same time providing patients with a realistic view of what we are trying to achieve.

## Figures and Tables

**Table 1:** Quality rating of included studies

	**Grunfeld (1996)**	**Gulliford *et al* (1997)**	**Brown *et al* (2002)**	**Koinberg *et al* (2004)**	**Baildam *et al* (2004)**	**Kokko *et al* (2005)**	**Grunfeld *et al* (2006)**
Is the population under study defined (with inclusion and exclusion criteria)?	Yes	Yes	No (exclusion criteria omitted)	Yes	No	Yes	Yes
Are the main prognostic factors defined (at least age of patient and stage of tumour)?	Yes	Yes	Yes	Yes	No	Yes	Yes
Is treatment of first tumour specified?	Yes	Yes	Yes	Yes	No	Yes	Yes
Has a power calculation been carried out to assess the required cohort size?	Yes	No	No	Yes	No	No	Yes
Is cohort size sufficient for primary outcome measure (i.e. greater than any calculated sample size or of sufficient size to detect clinically significant difference)?	Yes	Yes	No	Yes	Yes	Yes	No
Is a comparison made of baseline characteristics (age and stage) between participants and those who refuse to participate?	Yes	Yes	Yes	No	No	No	No
Is mean or median follow up greater than 5 years?	No	No	No	Yes	Not given	No	No
Is loss during follow up specified?	Yes	No	Yes	No	No	No	No
Is the follow up schedule (including mammographic interval) specified?	Yes	Yes	No	Yes	No	Yes	Yes
Is mammographic frequency identical between follow-up groups?	Yes	Yes	Yes	Yes	Not given	Yes	Yes
Are mostly objective or validated outcomes used?	Yes	No	Yes	Yes	Yes	Yes	Yes
Were outcomes prospectively assessed?	Yes	Yes	Yes	Yes	Yes	Yes	Yes
Was the article published in peer reviewed journal?	Yes	Yes	Yes	Yes	No	Yes	Yes
Total score	12	9	8	11	3	9	9

**Table 2:** Characteristics of included studies

**Study**	**Year**	**Patient group**	**Percentage randomisation**	**Age**	**Primary therapy**	**Study design**	**Main outcome measures**	**Follow-up schedule**	**Duration of study or median follow up**
Grunfeld	1996	Two hundred and ninety-six primary operable patients attending routine follow up at all stages of follow up and free of metastatic disease	66.5%	Mean 59.1 GP and 62.4 hospital (s.d. 10.3 and 12)	53% mastectomy, 47% Breast Conservation (BCT)	Randomised controlled trial of follow up in general practice *vs* hospital.	Quality of life as measured by several validated questionnaires. Number of recurrences. Number of deaths. Time to diagnose recurrence from onset of symptoms. No data on survival	3 monthly clinical examination and history during the first year, 6 monthly for 4 years, then annual in one hospital, with 3 monthly first year, 4 monthly second year, 6 monthly for 5 years and then annual in the other. General practice group as per hospital of diagnosis. One to two yearly mammograms.	18 months
Gulliford	1997	One hundred and ninety-six primary operable patients attending routine follow up over a 24-month period at all stages of follow up and free of metastatic disease	93%	56 <49 years, 96 =50–65 years and 41 >65 years	68% BCT, 32% mastectomy	Comparison of frequent follow up *vs* annual follow up	Acceptability of randomisation and overall satisfaction. Interim use of telephone and general practioner. No data on survival or quality of life	3 monthly clinical examination and history during first year, 4 monthly second year, 6 monthly for 5 years and then annual. One to two yearly mammogram, depending on whether mastectomy or BCT in the control group, annual clinical examination, history and mammogram in the trial group	Median 16 months
Brown	2002	Sixty-one primary operable patients attending routine follow up at all stages of follow up and free of metastatic disease	50%	Mean 63 standard clinic, 68 in patient initiated	66% BCT, 34% mastectomy	Traditional clinic follow up *vs* patient-initiated follow up	Quality of life using validated questionnaires and satisfaction using unvalidated structured interview. NO data on survival	4–6 monthly clinical examination and history for first 5 years then annual in control group *vs* on request only in the study group. Annual mammograms in both	12 months
Koinberg	2004	Two hundred and sixty-four primary operable patients attending routine follow up at all stages of follow up and free from metastatic disease	Not given	58.8 (s.d. 10.1) in traditional group, 60 (s.d. 10.3) in the on demand group	84% BCT, 16% mastectomy	Traditional clinic follow up *vs* follow up on demand coordinated by a breast care nurse	Quality of life and satisfaction using validated questionnaires. Number of contacts with health professionals. Number of events and survival	3 monthly clinical examination and history for 2 years, 6 monthly for 3 years then annual for 5 years and annual mammogram in traditional follow-up group, with appointments on demand only and annual mammograms in the nurse-led follow up on demand group	5 years
Baildam	2004	Five hundred and twenty-five primary operable patients attending routine follow up at all stages of follow up and free of metastatic disease	78%	Not given	Not given	Comparison of standard follow up with those by hospital doctors or specially trained breast care nurses	Number of events Anxiety by validated questionnaire. Satisfaction using validated questionnaire. Economic comparison NO survival comparison	Not given, but identical for both arms	Not given
Kokko	2005	Four hundred and seventy-two primary operable patients attending routine follow up at all stages of follow up and free of metastatic disease	Not given	56.9 in frequent follow up group, 60 in infrequent group	54% mastectomy, 46% BCT	A comparison of 3 *vs* 6 monthly follow up (and of intense *vs* as required investigations)	Event detection and cost per event detected No data on survival	3 *vs* 6 monthly clinical examination and history.	Median 4.2 years
Grunfeld	2006	Nine hundred and sixty-eight women between 9 and 15 months after diagnosis of early-stage breast cancer, who had completed treatment and were disease free	55%	Mean 60.9 (in both groups)	73% BCT, 20% mastectomy and 7% biopsy only	A comparison of follow up by general practioner *vs* hospital follow up	Quality of life using validated questionnaires. Significant clinical events (metastases related). Number of local recurrences and deaths	3–6 monthly for 3 years, 6 monthly for 2 years then annual, with annual mammogram	Median 4.5 years from diagnosis (3.5 from randomisation)

Abbreviations: BCT=breast conservation; s.d.=standard deviation.

**Table A1:** 

1	Adult/
2	‘Aged 80 and over’/ or aged/ or middle aged/
3	(neoplasm$ or cancer$ or tumour$ or tumour$ or malign$ or carcinoma$ or adenocarcinoma$ or sarcoma$ or dcis or ductal or infiltrat$ or intraduct$ or lobular$ or medullary$).mp.
4	breast.mp.
5	3 and 4
6	(follow up or follow-up or recurrence).mp. [mp=ti, ab, tx, kw, ct, ot, sh, hw]
7	1 and 2 and 5 and 6
8	Quality of life.mp. or ‘Quality of Life’/
9	patient satisfaction.mp. or Patient Satisfaction/
10	survival rate.mp. or Survival Rate/
11	8 or 9 or 10
12	7 and 11

**Table A2:** 

1	Adult/
2	‘Aged 80 and over’/ or Aged/ or Middle aged/
3	(neoplasm$ or cancer$ or tumour$ or tumour$ or malign$ or carcinoma$ or adenocarcinoma$ or sarcoma$ or dcis or ductal or infiltrat$ or intraduct$ or lobular$ or medullary$).mp.
4	breast.mp.
5	3 and 4
6	(follow up or follow-up or recurrence).mp. [mp=ti, ab, tx, kw, ct, ot, sh, hw]
7	1 and 2 and 5 and 6
8	Quality of life.mp. or ‘Quality of Life’/
9	patient satisfaction.mp. or Patient Satisfaction/
10	survival rate.mp. or Survival Rate/
11	8 or 9 or 10
12	7 and 11
13	11 and randomised

**Table A3:** 

1	TS=(neoplasm* or cancer* or tumour* or tumor* or malign* or carcinoma* or adenocarcinoma* or sarcoma* or dcis or ductal or infiltrat* or intraduct* or lobular* or medullary*)
2	TS=breast
3	1 and 2
4	TS=(follow up or follow-up or recurrence)
5	1 and 2 and 4
6	1 and 2 and 4 and randomised

## Appendix A

Search string used to identify relevent studies Table A1

## Appendix B

Search string used to identify relevent studies in CancerLitTable A2

## Appendix C

Search string used to identify relevent studies in Web of SciencesTable A3
